# An Essay on Quackery, Read before the Atlanta Medical Society, and Published by That Body

**Published:** 1856-05

**Authors:** T. C. H. Wilson

**Affiliations:** Atlanta, Ga.


ATLANTA

### Medical and Surgical Journal.

Vol. I.] MAY, 1856. [No. 9.

### ORIGINAL COMMUNICATIONS.

### ARTICLE I.

*An Essay on Quackery, read before the Atlanta Medical Society, and published by that body.* By T. C. H. Wilson, M. D., Atlanta, Ga.

The subject upon which I wish to offer a few irregular thoughts, is one which has, from time immemorable, called down the most abusive epithets of the profession; which has of late, in this fast, this young American age, in which Doctors are made as if by magic, from every class, gender and sex, has called out invective, the most opprobrious, in fact, which has almost beggared language for terms sufficiently reproachful to express the deep disgust, the bitter chagrin of our profession.

I claim to have as much contempt, to have as deep, as irreconcilable, as unmitigated animosity against quacks and quackery, "*et id omne genus*," as any other man in or out of the profession. I do not intend, Mr. President, (indeed this is not the time and place,) to abuse quackery. It is to the treatment and prognosis of the monster, of the hydra-headed beast, I wish to call your attention. If you disagree with me, please "pass my imperfections by," and remember that this is merely the opinion of one of the rank and file of the profession.

I do not, sir, I cannot, condemn so much as some others have, the man who is practising a speciality, or who has scattered broadcast his nostrum throughout the land, when I consider that his is a business, a one-idea transaction; he has but one object in view, but one end to obtain, to wit: pecuniary emolument; and see, sir, with what ease, with what speed, he attains the end desired. Look at those princely fortunes so soon acquired; at that massive pile erected to protect himself and family from the ruthless storm; at that splendid equipage to convey him whither he wishes to go; and tell me, sir, why is this? Where is the cause, and what is the remedy? In my opinion, they are all with us. The causes originated with us. The remedy is in our hands. We have been remiss; we have been wofully negligent in our duty, to ourselves and the public. To ourselves, by permitting, by inviting, ignorant one-idea money men to enter the profession, clothe them with the appellation of Doctor, and armed with a Diploma, they have gone forth thus equipped, and practised a speciality, or promulgated a nostrum, and obtained their end—money. Others, witnessing their success, have followed suit; and thus the world has become deluged with quacks, nostrums, &c. This has been, and now is, the great cause of quackery. This we can remedy by guarding more closely the portals to the green room, or by withdrawing the Diploma from the renegade of his *alma mater*. Pardon me; I do not wish to be dictatorial.

We have been remiss in our duty to the public. The public are ignorant; they are wofully ignorant relative to our science. "Mystery is the mother of obedience to my mandates," said the Priest of the dark ages. Mystery is the mother of obedience to my orders, reiterates the Doctor of the nineteenth century. There has always been too much mystery, too many wise looks, too many high-sounding, unintelligible phrases wrung into the ears of people. This has been done until every thing medical is stamped with mystery. This has been done until the people look for, they expect, they almost demand, a mysterious prescription—a compound that is good for everything in general, and most diseases in particular; and here it is the unprincipled mountebank steps in to supply the mysterious demand: and this he does by printing cards, circulars, almanacs, and books for parlor-table ornaments and popular reading. The other professions understand this want of knowledge among the masses, and have been industrious in supplying them. The clergy, for instance; they not only have seminaries to educate their preachers, and libraries, journals, synods, conferences, associations and assemblies, to keep them in good order, but they publish books, periodical, and papers, for popular reading. They battle their enemies in open controversy, and baffle them with silent influences by the fire side. Therefore it is, that Anglosaxons of mighty intellect are dictating religion to the world, while Indians and old women in pantaloons are dictating its medicine. Then why not emulate their example; for how is a plain, common man to decide who he shall send for when advice is needed? He knows not whether to send after Dr. Lobelia, Dr. Hydropath, Dr. Homeopath, Dr. Alopath, Dr. Urine Squirter, or Dr. Table Tipper, for all are called Doctor; and that is all he knows about them. Which of them knows any medical truth, or whether any of them do, is altogether a doubtful point in his mind; he does not know what to decide. How can he? He has had no opportunity furnished of knowing. Yet, he must have medical advice. He betakes himself to reading, or inquires of some friend who has read, who he shall send after. He reads, perhaps, a copy of the water-cure journal that settles his opinion; another picks up a medical almanac, or a patent medicine pamphlet, and that settles his faith; another pins his hopes to a newspaper advertisement. If the first selection does not meet his expectations, (and it seldom does,) the next necessity for medical advice, he tries another; and thus he goes through the whole routine of quackery before he hits the truly scientific physician. But! exclaims the Doctor, I will not submit my sanctified thoughts to their profane gaze. Then, sir, if you will not, sit quietly in your office, look dignified, speak mysteriously, and see the wily charlatan do your legitimate business, and go swiftly to fame and fortune.

If our profession would do as other professions have done, it is my prognosis that medicine would take her proper stand among the professions, and quackery would vanish before the light of pure science as fog before the sun. But, until then, in my opinion, she is doomed to remain stationary. Stationary! did I say? No, sir, retrograde; for science is never stationary; she is always advancing or receding. Then let us cultivate true science, for true science is not mysterious. The more scientific a man is, or the more thoroughly he is acquainted with any pursuit, the less mysterious it is, and the more capable he is to explain intelligibly all that is misunderstood by the uninitiated; and a proper knowledge of who and what is scientific in medicine, is, in my humble opinion, the great desideratum to the successful uprooting and destruction of the whole family of quacks of every name, country, and color; and then, and not till then, will our profession take that high and legitimate position for which "good men and true" have labored for centuries to obtain.

